# Designing and Evaluating a Personalized, Human-Centered Dietary Decision Support System for Use Among People With Diabetes in an Indian Setting: Protocol for a Quasi-Experimental Study

**DOI:** 10.2196/13635

**Published:** 2022-03-08

**Authors:** Dinesh Kumar, Ashok Bhardwaj, Shruti Sharma, Bhavya Malhotra, Chioma Amadi-Mgbenka, Ashoo Grover, Ashish Joshi

**Affiliations:** 1 Department of Community Medicine Himachal Pradesh India; 2 Dr. Radhakrishnan Government Medical College Himachal Pradesh India; 3 Foundation of Healthcare Technologies Society New Delhi India; 4 Graduate School of Public Health and Health Policy City University of New York New York, NY United States; 5 Indian Council of Medical Research New Delhi India

**Keywords:** type 2 diabetes, diabetes management, dietary decision support, diet record, India

## Abstract

**Background:**

Human-centered dietary decision support systems are fundamental to diabetes management, and they address the limitations of existing diet management systems.

**Objective:**

The objective of the proposed study is to evaluate the use of an interactive, telephone-linked, personalized, human-centered decision support system for facilitating the delivery of personalized nutrition care for patients with diabetes.

**Methods:**

A quasi-experimental trial was conducted between the period of June and December 2018. Study participants were recruited from Community Health Center, Dharamshala, Kangra (urban population), and Model Rural Health Unit, Haroli Block, Una (rural population). Eligible participants included adults aged ≥30 years with controlled or uncontrolled diabetes, those who agreed to participate in the study, those who were available for follow-up interviews, and those with a telephone or computer at home. Diabetic status was determined via a physician’s diagnosis. Individuals with mental or physical challenges that affected their ability to use an electronic diet record, those who were not available for a telephone follow-up, and those who were involved in other protocols related to dietary assessments were excluded. The study participants were randomized into the following two groups: the intervention group (telephone-linked dietary decision support system) and the control group (paper-based diet record). Study participants in the intervention group recorded their daily dietary intake by using a telephone-linked, personalized, human-centered dietary decision support system and received personalized feedback and diet education via SMS text messaging. Study participants in the control group were provided with only a paper-based diet record for documenting their daily dietary intake. Follow-up visits were conducted at 3 and 6 months from the baseline in both groups. Differences in diabetes knowledge, attitudes, and practices will be measured across groups.

**Results:**

The collection of baseline data from 800 study participants in both the intervention (n=400) and control groups (n=400), which were stratified by urban (control group: n=200; intervention group: n=200) and rural settings (control group: n=200; intervention group: n=200), has been completed. Follow-up data collection for months 3 and 6 is ongoing and is expected to be completed by October 2019.

**Conclusions:**

We anticipate that the intervention group will show significant changes in nutrition knowledge, attitudes, and practices; satisfaction with care; and overall diabetes management. We also expect to see urban-rural differences across the groups. The uniqueness of our nutrient data capture process is demonstrated by its cultural and contextually relevant features—diet capture in both English and Hindi, diet conversion into caloric components, sustained diet data collection and participant adherence through telephone-linked care, and auto-generated reminders.

**International Registered Report Identifier (IRRID):**

DERR1-10.2196/13635

## Introduction

Type 2 diabetes (T2D) is a rapidly growing chronic health problem, and the complications of T2D cause significant morbidity and mortality. Globally, 415 million people are living with T2D mellitus, and this is estimated to increase to 642 million people by 2040 [1]. The proliferation of T2D is the most notable in low- and middle-income countries, and this has been attributed to a number of factors, including an aging population, population growth, urbanization, the increasing prevalence of obesity, and physical inactivity [2-5]. India, China, and the United States have the highest diabetes incidence rates globally. According to the International Diabetes Federation estimates, the number of patients with diabetes in India is projected to increase to approximately 70 million by 2025—almost double the amount from 2007 [1].

A key outcome of proper diabetes management is the development of healthy eating patterns [3]. In addition, daily physical activity sessions are recommended to regulate blood glycemic levels. [4]. Proper glycemic control is critical to the prevention or delay of the onset of acute and chronic complications and to the improvement of quality of life among people with diabetes. Providing self-management education and support tools to improve diabetes knowledge, foster treatment adherence, promote lifestyle changes, and enable the self-monitoring of blood glucose is fundamental to this process [5,6].

Dietary intake assessment is a fundamental step in developing interventions for people living with T2D [6]. Dietary intake has been measured by using a variety of methods in the existing literature, including dietary recalls, weighed diet records, diet history reports, and food frequency questionnaires [6-8]. None of these methods have demonstrated adequate accuracy and reliability in determining food intake. A variety of technology-based applications have also been developed for estimating and recording food consumption, but significant limitations have been reported, including small sample sizes, insufficient outcome evaluation periods, high costs, and inappropriate message framing [9,10]. Future studies using randomized controlled trial designs, larger sample sizes, and longer durations of evaluation are recommended to establish the intervention capabilities of technology-based diet interventions [10-14].

The objective of the proposed study is to evaluate the use of an interactive, telephone-linked, personalized, human-centered decision support system for facilitating the delivery of personalized nutrition care for patients with diabetes. To the best of our knowledge, this is the first study to design and develop an intervention model after assessing the needs of users from urban and rural settings in an Indian context. The proposed study has three specific aims, as follows:

- Examine and compare the factors that influence the intake of a healthy diet among people with diabetes living in the urban and rural settings of Himachal Pradesh
- Identify the necessary components of the personalized, human-centered dietary decision support system that can gather, analyze, and provide individualized dietary feedback
- Compare the effectiveness of the personalized, human-centered dietary decision support system to that of other paper-based methods of diet recording for documenting nutrient intake among people with diabetes

## Methods

### Ethics Approval

Ethical approval was obtained from the Institutional Review Board of Dr. Rajendra Prasad Medical College Kangra (institutional review board number: 116/2016).

### Study Design

**Figure 1:**
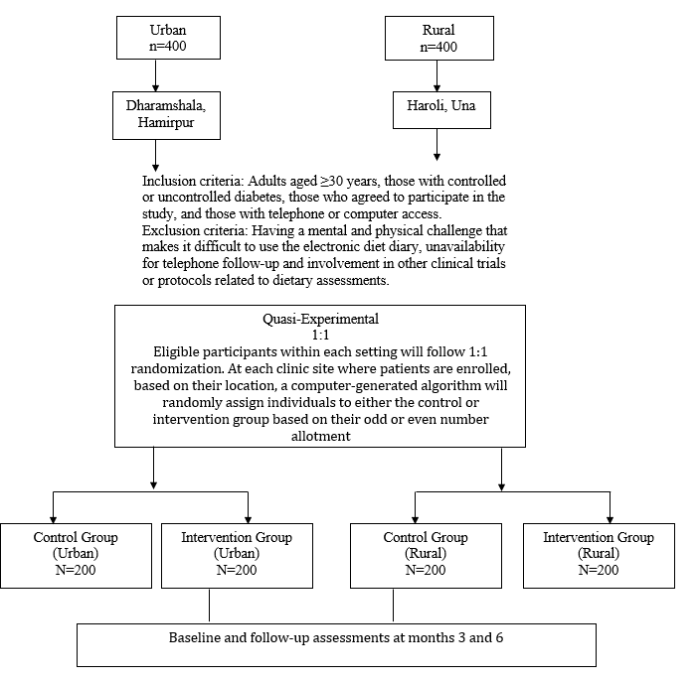
Study participant recruitment.

A quasi-experimental trial was conducted between the period of June and December 2018 (Figure 1). Study participants were recruited from the following two urban and rural locations: Community Health Center, Dharamshala, Kangra (urban population), and Model Rural Health Unit, Haroli Block, Una (rural population). Eligible study participants included (1) adults aged ≥30 years, (2) those with controlled or uncontrolled diabetes, (3) those who agreed to participate in the study, (4) those who were available for follow-up interviews, and (5) those with a telephone or computer at home. Diabetic status was based on a physician-confirmed diagnosis of diabetes and the prescription of medications for diabetes management. The exclusion criteria comprised the following: (1) the presence of any mental or physical challenge affecting the study participants’ ability to use an electronic diet record, (2) unavailability for a telephone follow-up, and (3) involvement in other clinical trials or protocols related to dietary assessments. The study was funded by the Indian Council of Medical Research.

### Study Groups

The study participants were randomly assigned into the following two groups: the intervention group (telephone-linked dietary decision support system) and the control group (paper-based diet record; Figure 1). The randomization was conducted following the assessment of participants’ eligibility for inclusion in the study. Study participants in the intervention group (group 1) recorded their daily dietary intake by using a telephone-linked, personalized, human-centered dietary decision support system and received personalized feedback and diet education via SMS text messaging. The personalized, human-centered dietary decision support system is a telephone-linked decision support system designed to provide tailored dietary education to patients with diabetes. The study participants were able to access the personalized, human-centered dietary decision support system through their computers, cell phones, or a telephone-linked service, depending on the technology platform available to them. The telephone-linked service enabled the participants (including those who did not have a technology platform to access the personalized, human-centered dietary decision support system) to receive phone calls from our research team in order to record their daily dietary intake. The personalized, human-centered dietary decision support system also generated automatic alerts that served as reminders to the participants who had not reported their daily diet intake. Study participants in the control group were provided with a paper-based diet record for documenting their daily dietary intake. Both the intervention group and control group were provided with a diabetes educational booklet at baseline. However, the study participants in the control group were also provided with a booklet to document their diet log on a daily basis. Follow-up visits were conducted at 3 and 6 months from the baseline in both groups. Baseline data were gathered on sociodemographics; health literacy; physical activity; anthropometric measurements; blood sugar testing; and diabetes knowledge, attitudes, and practices (KAPs). Follow-up data were gathered on anthropometric measurements, diabetes KAPs, blood sugar testing, and satisfaction with medical care.

### Intervention Development

**Figure 2:**
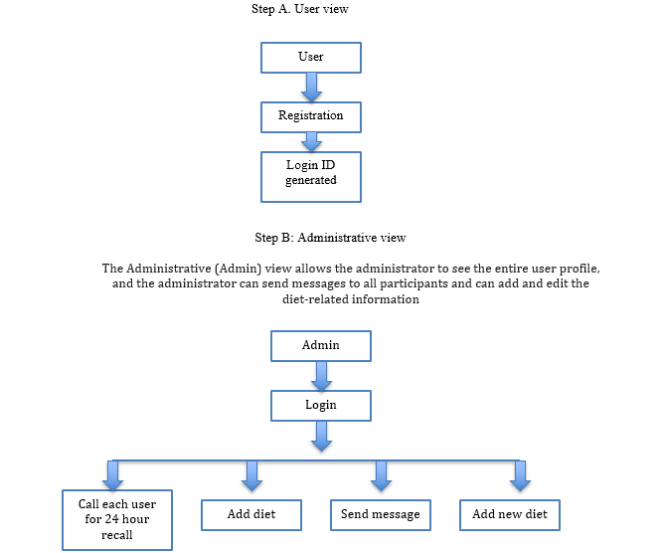
Dietary decision support system workflow.

**Figure 3:**
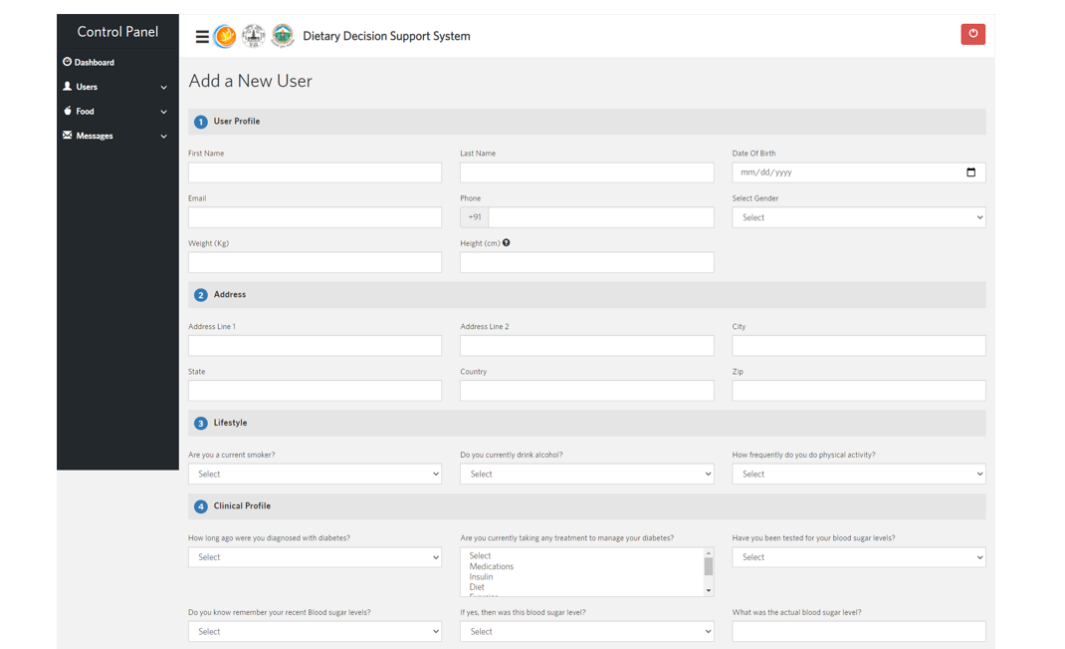
Administrative control panel: entry of study participant demographics.

**Figure 4:**
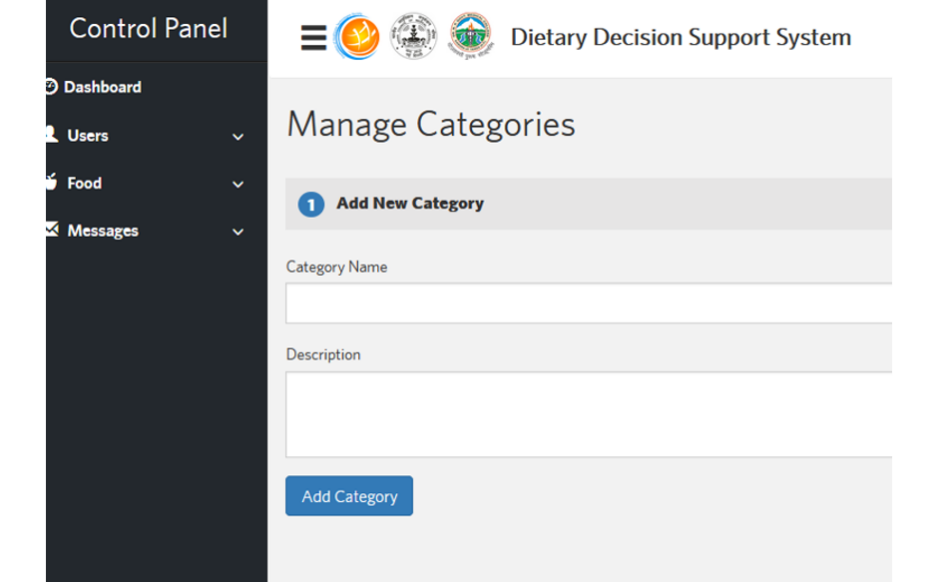
Administrative control panel: addition of food categories.

A telephone-based, personalized, human-centered dietary decision support system for populations with diabetes was designed to provide tailored dietary education to patients with diabetes (Figure 2). A human-centered approach was used in the design process. Human-centered design principles require end users to be prioritized in intervention design and development [15]. User characteristics, needs, and preferences are vital for ensuring the optimal use of technology-enabled interventions [15] (Figure 3). The failure to fully implement human-centered design in dietary intervention designs has been a limitation in prior studies [11]. The personalized, human-centered dietary decision support system is comprised of the following components: (1) the electronic monitoring of diet records, (2) personalized dietary and disease feedback, (3) the nutrient calculation of daily diet intake per meal, and (4) alerts and reminders (Figure 4).

**Figure 5:**
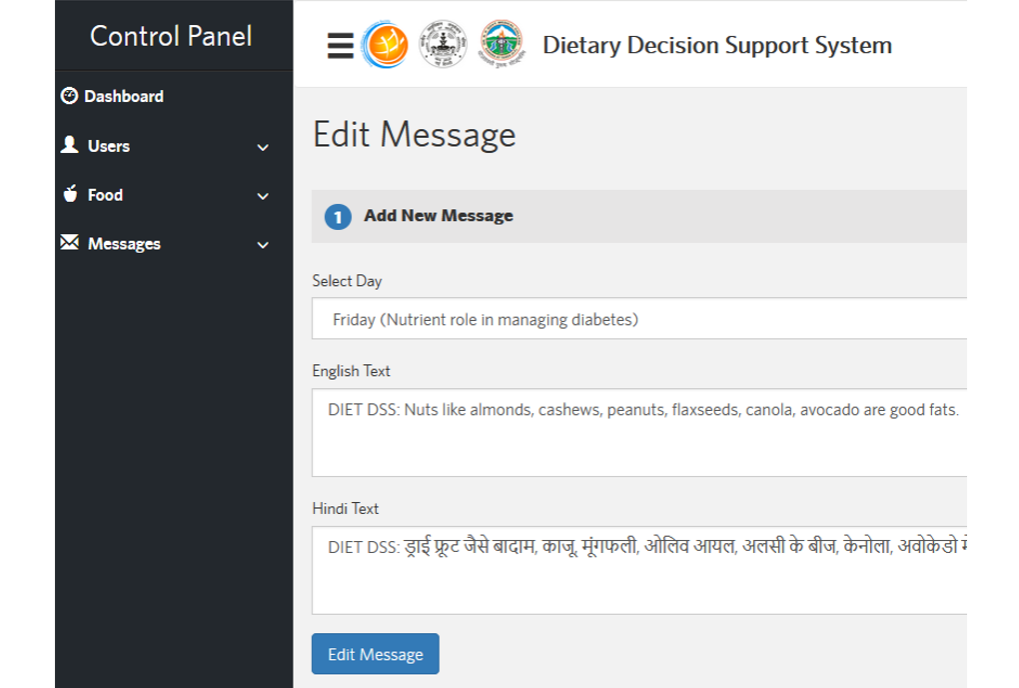
Administrative control panel: generation of personalized dietary feedback.

The personalized, human-centered dietary decision support system can be used on cell phones, PDAs, and computers, depending on the technology platforms available to users across various Indian settings. Dietary information from the prospective users was gathered on a daily basis. This information included the various food sources consumed and the quantity and timing of food consumption. A nutrient database of food choices and their caloric values was subsequently created [16]. This nutrient database included the following components: food type, food class, quantity, and calorie estimation. A message library in both the English and Hindi languages (a local Indian dialect) was prepared, so that individuals could be sent weekly messages on how best to manage their diet. Based on the study participants’ dietary intake, interactive tailored feedback was provided (Figure 5). Study participants also received auto-generated reminders and alerts if their dietary information was not received as scheduled.

### Study Enrollment and Data Collection Procedure

Data were collected at baseline, and follow-ups were conducted at months 3 and 6, resulting in 4 data collection time points. After each time point, the field-workers scheduled follow-up interviews to administer the study questionnaires. The study staff who were collecting data were blinded to the group assignments.

### Informed Consent

Informed consent forms were administered to the eligible individuals, and those who consented were enrolled in the study. The institutional review board–approved consent forms were administered by members of our research team to the eligible individuals. These forms described the study, the measures used by the researchers to protect the confidentiality of the responses, and the voluntary nature of the study. The consent forms were countersigned by members of the research team, and copies were provided to the study participants.

### Data Entry and Quality Assurance

Data entry was performed by a team of field-workers and data management personnel. To ensure efficiency and high-quality data collection and processing, we (1) used a well-trained team of field-workers, (2) used a clearly defined study manual, (3) conducted weekly meetings with the research team, and (4) maintained logs of all patient contacts. To ensure efficient and accurate data management, we maintained (1) logs of all of the data instruments that were filled during each visit for every patient, (2) central data processing, and (3) weekly data checks. Data security was ensured through regular backups, password protection, and storage in a locked file cabinet.

### Variables Assessed

#### Sociodemographics

Baseline data were gathered on study participants’ age, income level, employment status, education level, smoking status, and alcohol consumption. Information was also collected on computer usage, internet usage, the frequency of computer and internet usage, and sources of health information.

#### Health Literacy

Health literacy is defined as “the ability to perform basic reading and numerical tasks required to function in the health care environment” [17]. Health literacy was assessed by using a 3-item health literacy screening questionnaire [17]. The questions included the following: (1) “How often do you have someone (like a family member, friend, hospital/clinic worker or caregiver) help you read hospital materials,” (2) “How often do you have problems learning about your medical condition because of difficulty understanding written information” (problems reading), and (3) “How confident are you filling out forms by yourself” (confident with forms)? Responses were rated on a Likert scale ranging from 0 to 4 and included the following options: “all of the time,” “most of the time,” “some of the time,” “a little of the time,” and “none of the time” [17].

#### Anthropometry

Height, weight, and waist circumference were measured by using a standard technique [18]. BMIs were computed from the height and weight measurements.

#### Physical Activity Assessment

All participants completed a validated short form of the International Physical Activity Questionnaire in order to calculate the total time that they spent on performing various forms of physical activity—recreational activities, occupational activities, household work, and transportation-related activities—in the last 7 days [19]. Total weekly physical activity—metabolic equivalents of task (METs; MET hours per week)—will be calculated as the weighted sum of the reported time spent at each intensity by using a MET value specific to each category (walking: 3.3 METs; moderate: 4 METs; vigorous: 7 METs) [19].

### Outcomes

The study outcomes include dietary KAPs. These outcomes were assessed by using the following tools. Satisfaction with medical care was also assessed.

#### Nutrition Knowledge Scale

The Nutrition Knowledge Scale comprises the following four types of items: (1) 10 items on the relationship between diet and disease, (2) 10 items on food comparisons in terms of their nutrient content (eg, fat, fiber, calcium, calories, and sodium); (3) 6 items on the daily serving requirements of different food groups; and (4) 5 items on weight and weight loss. The scale is in a multiple-choice format, and 1 point is awarded for correct answers; otherwise, 0 points are awarded. The interitem reliability (Cronbach α coefficient) of the Nutrition Knowledge Scale is .78 [20].

#### Nutrition Attitude Scale

The Nutrition Attitude Scale consists of 19 items and the following three sections: (1) care about nutrition (8 items), (2) emotional eating (6 items), and (3) the importance of nutrition (5 items). The response options are rated on a 5-point Likert scale and include “strongly disagree,” “disagree,” “neutral,” “agree,” and “strongly agree.” Reverse sentence items are scored reversely. The Cronbach α coefficient of the Nutrition Attitude Scale is .73 [20].

#### Nutrition Behavior Scale

The Nutrition Behavior Scale is a 24-item scale with responses on a 5-point Likert scale. It comprises the following two sections: (1) food selection and care about nutrition (15 items) and (2) emotionally and externally cued eating (9 items). The response items include “never,” “seldom,” “sometimes,” “often,” and “always,” ranging in score from 1 to 5. Reverse sentence items are scored reversely. The Cronbach α coefficient of the Nutrition Behavior Scale is .76 [20].

#### Satisfaction With Medical Care

Satisfaction with medical care will be measured by using the Client Satisfaction Questionnaire-8 (CSQ-8). The CSQ-8 is an 8-item questionnaire in which items are rated by using a 4-point Likert scale [21].

#### Blood Sugar Testing

We will also assess participants’ blood sugar levels at baseline and at 6 months to assess the pattern of blood sugar control.

### Data Analysis Plan

In the first step of the exploratory analysis, we will present a table to summarize the overall characteristics of all variables, including outcome, confounding, and process variables. This table will also serve as quality control for the original data and be used to mine missing data patterns and outliers. The distributions of the continuous outcomes will be explored for normality, and transformations will be used if necessary. Continuous variables will be summarized using means, medians, SDs, and ranges, while categorical variables will be examined by using frequencies and percentages. An exploratory analysis on process variables will be used for a post hoc analysis. As a third step, we will cross-tabulate the group assignments against outcome variables to assess the association in the initial stage of the study.

### Sample Size Justification

The sample size calculation was based on knowledge, attitude, and behavior outcomes, as reported in previous studies [22]. To detect a mean change of 0.2 with an SD of 0.7 at a 2-sided .05 α level of significance and a power of 85%, a sample size of 200 individuals in each group is needed. The total participant sample size will be 800.

### Statistical Analysis

Baseline characteristics will be summarized and stratified by intervention group. The primary outcomes—adherence to dietary guidelines, KAPs, and patient satisfaction with medical care—will be compared between groups by using a log-rank test. A Cox proportional hazards regression model will be used to adjust for confounders that differ significantly between the two groups at the .20 level of significance. A generalized linear mixed model using a binomial distribution and logit link function will be used to examine the binary secondary objective—subjects’ satisfaction with medical care. The group types (ie, the intervention and control groups) will be used as a fixed effect, and subject data will be used as random effects. This will be done by using a variance components covariance structure. To adjust for confounders that differ significantly between the two groups at the .20 level of significance, covariates will be added to the model, and a linear regression model will be used.

## Results

Data collection was conducted between the period of June and December 2018. The collection of baseline data from 800 study participants in both the intervention (n=400) and control groups (n=400), which were stratified by urban (control group: n=200; intervention group: n=200) and rural settings (control group: n=200; intervention group n=200), has been completed. Follow-up data collection for months 3 and 6 is ongoing. We expect the follow-up data collection to be completed by October 2019. We are currently analyzing the baseline data and generating reports, which will be presented in our upcoming manuscript.

## Discussion

We anticipate that our results will show a significant difference in nutrition KAPs between the study groups. The study participants receiving tailored dietary education will likely show better improvements in their KAPs and overall diabetes management. We also anticipate that satisfaction with care (measured by the CSQ-8) will be higher in the intervention group, owing to the unique features of our interactive, telephone-linked, personalized, human-centered decision support system (eg, personalized dietary education and reminder alerts for completing the food log). We also expect to see urban-rural differences across the intervention and control groups. Our dietary decision support system is the first of its kind to be successfully implemented in a lower-middle–income country such as India, and it has the potential to inform dietary data management on a wider scope. Key gaps in diet-related interventions are centered on the tailoring, sustainability, and evaluation of such interventions [15,23]. Studies that were conducted to examine the effectiveness of existing diet apps highlighted the need for tailoring approaches that can address the personal needs of the users [23]. Another crucial gap in the design approaches of existing diet apps is the challenge of identifying which specific components are associated with intervention effectiveness. The task of identifying specific components associated with effectiveness becomes an arduous one when human-centered approaches are not effectively incorporated into the design process. A systematic review investigating the effectiveness of eHealth and mobile health interventions that promote physical activity and healthy diets across 13 low- and middle-income countries showed that it was not possible to identify the specific components associated with app effectiveness among the included studies [23]. A human-centered design approach is needed to address these challenges. The uniqueness of our nutrient data capture process is demonstrated by its incorporation of human-centered, cultural, and contextually relevant features—diet capture in both English and Hindi, diet conversion into caloric components, sustained diet data collection and participant adherence through telephone-linked care, and auto-generated reminders. We conducted focus groups with the study population in addition to literature reviews, with the aim of identifying user characteristics, needs, and preferences that potentially influence users’ satisfaction with and use of the interactive, telephone-linked, personalized, human-centered decision support system [24-26].

## Abbreviations

CSQ-8: Client Satisfaction Questionnaire-8
KAP: knowledge, attitude, and practice
MET: metabolic equivalent of task
T2D: type 2 diabetes
